# Dimephosphon Radioprotective Properties on the Model of Radiation Injury In Vivo

**DOI:** 10.32607/actanaturae.27662

**Published:** 2025

**Authors:** D. A. Kiseleva, M. A. Melchenko, O. I. Yarovaya, N. V. Basov, A. D. Rogachev, A. G. Pokrovsky, N. F. Salakhutdinov, T. G. Tolstikova

**Affiliations:** N.N. Vorozhtsov Novosibirsk Institute of Organic Chemistry, Siberian Branch of Russian Academy of Sciences, Novosibirsk, 630090 Russia; Novosibirsk State University, Novosibirsk, 630090 Russia

**Keywords:** radioprotective properties, radiation injury, Dimephosphon, metabolomic screening

## Abstract

Radiation therapy is a commonly used cancer treatment modality. However, its application is limited because of its toxicity to healthy tissue. The search for effective radioprotective agents remains one of the key goals of radiation oncology and radiobiology. This study focuses on experimental modeling of radiation injury in animals and the investigation of Dimephosphon radioprotective properties, a drug exhibiting anti-acidotic, antitumor, and antioxidant activities. It was shown that 14-day administration of the drug at a dose of 750 mg/kg after single-dose (5 Gy) irradiation of CD-1 mice resulted in a local radioprotective effect, reducing the severity of the radiation-induced injury to the intestinal epithelium and splenic capsule. The results of metabolomic screening revealed that the levels of the key metabolites responsible for antioxidant properties such as alpha-tocopherol, nicotinamide riboside, N-carbamoyl-L-aspartate, and adenylosuccinate were significantly increased, indicating that the Dimephosphon drug provides enhanced antioxidant protection.

## INTRODUCTION

Cancer remains one of the leading causes of mortality worldwide. As of 2023, the incidence rate of malignant neoplasms in Russia was more than 670,000 new cases; and this parameter was on the increase by 8.0% compared to 2022 [1]. Radiation therapy (RT) is an effective method for combatting malignancies. It is estimated that ~ 50% of cancer patients receive radiation therapy, whereas ~ 70% require this treatment, and in some cases radiation therapy is the sole cancer treatment option available [2]. The lack of selectivity toward cancer cells, which disrupts metabolic processes in healthy tissues and organs and results in severe complications, including radiation-induced injury, is the primary factor limiting broader application of RT [3]. The emergence of more selective RT modalities does not eliminate the toxicity to healthy tissues. Therefore, approaches that would combine radiation therapy and systemic administration of radioprotective agents are currently being investigated. Importantly, the radioprotective drugs approved for use in Russia are associated with serious adverse effects such as splenic rupture, acute respiratory distress syndrome, alveolar hemorrhage, and atrioventricular block, which limit the widespread application of these agents [4, 5, 6]. For this reason, the radioprotective potential of natural compounds is currently being extensively investigated; however, all the developments remain at the preclinical phase [7, 8]. Therefore, searching for effective low-toxicity agents protecting healthy tissues against radiation-induced damage during radiation therapy remains a critical challenge in radiation oncology and radiobiology.

To perform preclinical studies of novel radioprotective agents and optimize treatment strategies for different types of cancer, it is essential to be in possession of adequate animal models of radiation injury that would reliably and accurately replicate the key clinical manifestations and pathogenetic mechanisms of the disease in humans. The suitability of X-ray radiation with a peak voltage of 320 kV for inducing radiation injury on in vivo models has previously been demonstrated [9].

**Fig. 1:**
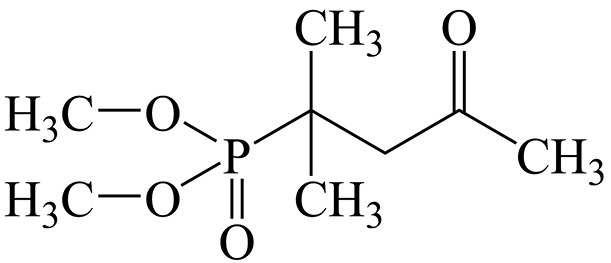
The structural formula of the active substance of Dimephosphon

We have developed a procedure for inducing radiation injury in laboratory animals (mice) that allows one to assess the efficacy and safety of radioprotective agents. The original drug Dimephosphon, manufactured in Russia, was selected as a radioprotective agent for validating this procedure. Dimephosphon is an aqueous solution of (1,1-dimethyl-3-oxobutyl)phosphonic acid dimethyl ester (Fig. 1); it is characterized by low toxicity and high bioavailability and can easily cross the blood–tissue barriers [10, 11].

In 1983, the drug (a 15% solution for oral and topical administration) was approved for clinical use as an anti-acidotic and vasoactive agent [12, 13, 14]. Later, the efficacies of three radioprotective compounds (sea buckthorn oil, Evdoshchenko oil solution, and drug Dimephosphon) were evaluated in the context of radiation therapy for laryngeal cancer. Administration of Dimephosphon resulted in the smallest quantitative differences in the laryngeal air column thickness, a key indicator of acute radiation syndrome, measured before the initiation of radiation therapy and after a 40-Gy dose had been administered [15].

## EXPERIMENTAL

**Animals**

All the manipulations with animals were conducted in strict compliance with the legislation of the Russian Federation, Decision No. 81 “On the Approval of the Rules of Good Laboratory Practice of the Eurasian Economic Union in the Field of Drug Circulation” dated November 3, 2016, and the provisions of Directive 2010/63/EU of the European Parliament and the Council of the European Union dated September 22, 2010 on the protection of animals used for scientific purposes. The study protocol was approved by the Bioethics Commission of the Laboratory of Pharmacological Research, N.N. Vorozhtsov Novosibirsk Institute of Organic Chemistry, SB RAS (Protocol No. R-14-2025-01-01 dated January 10, 2025).

Female outbred CD-1 mice (weight, 25–30 g) were procured from the SPF vivarium of the Institute of Cytology and Genetics, SB RAS, Russia. The animals were housed under optimal conditions (temperature, 21 ± 1.5°C; humidity, 40–60%; 12-h day/night cycle; ad libitum access to water and pelleted forage). Prior to the experiments, the mice were acclimatized to the housing conditions for 1 week.

**Compound under study**

Dimephosphon® (OJSC “Tatchempharmpreparaty”, Kazan, Russia) used in this study was administered intragastrically at a dose of 750 mg/kg (0.1 mL per 10 g of body weight). The animals received the first dose 3 h prior to irradiation; the drug was then administered as a single dose every 24 h during 14 days.

**Experimental design and setup**

An X-RAD 320 irradiation system (Precision X-Ray Inc., Branford, CT, USA) with a fixed distance from a radiation source (SSD 50 cm), equipped with a medium-hardness filter (0.75 mm tin, 0.25 mm copper, 1.5 mm aluminum), was used to experimentally induce radiation injury in mice. Total-body single-dose (5 and 7.5 Gy) irradiation of mice (n = 6) was performed at a dose rate of ~ 0.98 Gy/min. The survival rate of the animals was assessed on days 4, 7, 11, and 14 post-irradiation, and a radiation dose for studying the radioprotective effect of the drug was selected.

The radioprotective properties of Dimephosphon were assessed at the next stage in mice that had received a single selected radiation dose. The animals were randomly allocated into three groups (n = 8): mice in group 1 were administered 750 mg/kg Dimephosphon (DMPN); mice in group 2 were administered 750 mg/kg Dimephosphon + total-body irradiation (DMPN + IR); and mice in group 3 were subjected to total-body irradiation only (IR).

Animals’ body weight was measured prior to irradiation (point 0) and then on days 4, 7, 11, and 14. Blood samples were collected from the retro-orbital sinus to conduct metabolomic screening and hematology testing. All the mice were euthanized on day 14; their organs (the thymus, heart, lungs, liver, and spleen) were weighed to calculate the organ mass indices and further used for histological examination.

**Hematology testing**

Complete blood count was performed using a MINDRAY BC-2800 Vet automatic hematology analyzer (Shenzhen Mindray Animal Medical Technology Co. Ltd., China). Peripheral blood samples (20 μL) collected into vials containing a standard volume of isotonic diluent were used for testing. The total counts of leukocytes, erythrocytes, platelets, hemoglobin concentration, and hematocrit, were determined.

**Histological examination**

The collected organs (the thymus, heart, lungs, liver, and spleen) were weighed to calculate the organ mass indices. The spleen and small intestine were fixed in 10% neutral buffered formalin, dehydrated in ethanol and xylene of different concentrations using a MICROM automated system (Carl Zeiss, Germany). The tissue samples were then embedded in paraffin blocks. Sections (4 μm thick) were prepared using a rotary microtome and stained with Hematoxylin and Eosin (H&E). The prepared samples were examined by optical microscopy (×100 magnification), with Köhler illumination alignment. The AxioVision software was used to perform a morphometric analysis of the histopathological images and calculate the intervillous space, length of intestinal villi, and thickness of the splenic capsule for assessing the severity of organ damage.

**Metabolomic screening**

Sample preparation. Dried whole-blood spot samples were prepared for metabolomic analysis. A 10 μL aliquot of blood was applied to Whatman 903TM protein saver cards (GE Healthcare, #10534612, USA) and airdried at room temperature for 3 h. The samples were stored at –70°C until sample preparation, which was conducted in accordance with the protocol described in ref. [16]. The dried blood spots were resected from the cards, placed into 0.5 mL polypropylene vials, and 150 μL of a pre-cooled MeOH–ACN–H2 O mixture (40:40:20, v/v/v) was added. The samples were incubated at +4...+5°C for 20 min and centrifuged at 16,000 rpm (~ 24,000 g) on an Eppendorf 5417R centrifuge for 10 min at +4°C. The supernatant was transferred to plastic inserts for chromatographic vials and analyzed.

**Analysis of the samples**

The samples were analyzed by high-performance liquid chromatography–tandem mass spectrometric assay (HPLC-MS/MS) according to ref. [17]. Chromatographic separation was conducted on an LC-20AD Prominence chromatography system (Shimadzu, Japan) and an CTO-10ASvp column oven. The mobile phase consisted of eluent A (a 20 mM aqueous solution of ammonium carbonate adjusted to pH 9.8 with a 25% aqueous solution of ammonia, and 5 vol.% acetonitrile) and eluent B (100% acetonitrile). Each sample was analyzed twice: in the hydrophilic interaction liquid chromatography (HILIC) and reverse-phase chromatography (RPC) modes. The following conditions were used. HILIC gradient: 0 min – 98% B, 2 min – 98% B, 6 min – 0% B, 10 min – 0% B. The column was equilibrated for 4 min. RPC gradient: 0 min – 0% B, 1 min – 0% B, 6 min – 98% B, 16 min – 98% B. The column was equilibrated for 3 min. The flow rate in each analysis was 300 µL/min. Sample volume was 2 µL. In both chromatography modes, the analysis was conducted using a monolithic column (2 × 60 mm) based on 1-vinyl-1,2,4-triazole. The monolithic material of the column was synthesized according to ref. [18]: copolymerization of a mixture consisting of styrene/divinylbenzene/1-vinyl-1,2,4-triazole monomers at a 10 : 50 : 40 volume ratio was performed in a glass tube.

Mass-spectrometric detection was conducted using an API 6500 QTRAP mass spectrometer (AB SCIEX, USA) equipped with an electrospray ionization source. A total of 489 metabolites were detected in the multiple reaction monitoring (MRM) mode in the regions of positive and negative ionization with polarity switching. The key mass spectrometric parameters were as follows: voltage of the ion source (IS), 5,500 and −4,500 V for the positive and negative ionization, respectively; drying gas temperature, 475°C; gas in the collision-activated dissociation cell was set at “high”; pressure of the nebulizing gas (GS1), drying gas (GS2), and the curtain gas (CUR) was 33, 33 and 30 psi, respectively. The declustering potential (DP) was ±91 V, the entrance potential (EP) was ±10 V, and the collision cell exit potential (CXP) was ±9 V. The dwell time for each MRM transition was 3 ms. Instrument control and data acquisition were performed using the Analyst 1.6.3 software (AB SCIEX). The precursor-to-product ion transitions, metabolite names, fragmentation times, and the respective collision energies were adapted from refs [19, 20].

**Statistical analysis**

Statistical analysis was performed with the Statistica 10.0 software (StatSoft, USA). The data were tested for normality using the Kolmogorov–Smirnov test. The Student’s t-test was used for the normally distributed samples; the Mann–Whitney U test was employed for the nonnormally distributed samples. The results are presented as the mean ± standard error of the mean (M ± SEM) or the mean ± confidence interval for nonparametric samples. The differences were considered statistically significant at p < 0.05. The diagrams were plotted using the Seaborn library (Python) and the Origin software.

## RESULTS AND DISCUSSION

**Assessment of animals’ survival rate exposed to irradiation with 5 and 7.5 Gy doses**

**Fig. 2:**
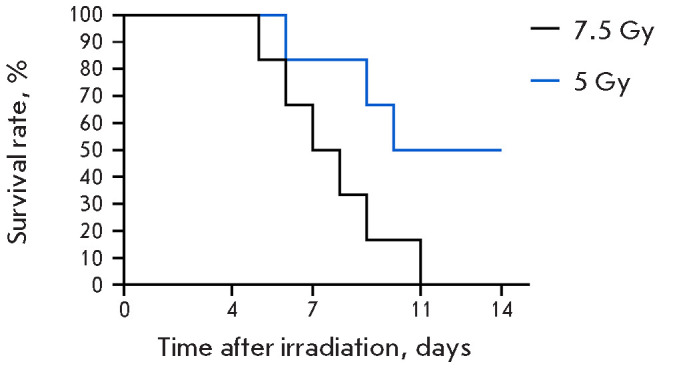
The survival rate of CD-1 mice after single-dose irradiation (5 and 7.5 Gy) (n = 6)

The optimal radiation dose for modeling radiation injury in experimental animals was determined at the first stage of the study. Total-body irradiation at a dose of 7.5 Gy caused 100% lethality on experiment day 11 (Fig. 2). Hence, the absolute lethal dose (causing death in 100% of mice) was identified; its further use was unreasonable. After single-dose (5 Gy) irradiation, 50% of mice remained alive by the end of the experiment (day 14), corresponding to the sublethal radiation dose (death in 50% of mice, LD_50_).

**The survival rate, mean body weight, and hematological parameters of mice exposed at a dose of 5 Gy radiation and administered Dimephosphon**

The effect of Dimephosphon oral administration on the organism of experimental animals subjected to single-dose (5 Gy) total-body irradiation was evaluated at the second stage of the study.

**Fig. 3:**
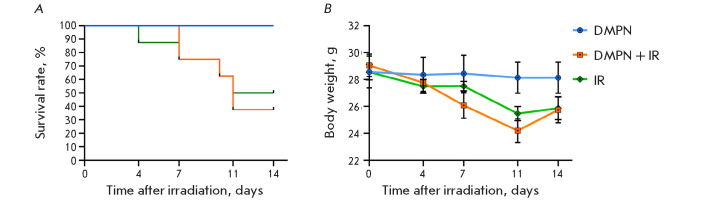
The effect of oral administration of 750 mg/kg Dimephosphon 3 h before 5 Gy total-body irradiation and daily after in CD-1 mice (n = 8, M ± SEM). (A) animal survival; (B) the mean body weight dynamics of the animals

The use of Dimephosphon did not increase the survival rate of the animals after exposure to X-ray radiation (Fig. 3A). On experiment day 14, the survival rate in the IR group was 50%, being ~ 40% in the DMPN + IR group. This was probably caused by the varying animal sensitivity to radiation [21], since the LD_50_ of the drug orally administered to mice was 3 g/kg [10]. Furthermore, the mean body weight of the irradiated animals was significantly reduced compared to the baseline, without statistical differences between the groups of irradiated (DMPN + IR, IR) and non-irradiated mice (DMPN) (Fig. 3B).

According to published scholarship, the development of radiation injury involves three syndromes: the hematopoietic (occurring at doses > 1 Gy), gastrointestinal (at doses of 6–15 Gy), and cerebrovascular (at doses > 20 Gy) ones. In other words, the hematopoietic system, spleen, thymus, and intestinal epithelium are first to sustain damage in response to exposure to ionizing radiation [22].

**Fig. 4:**
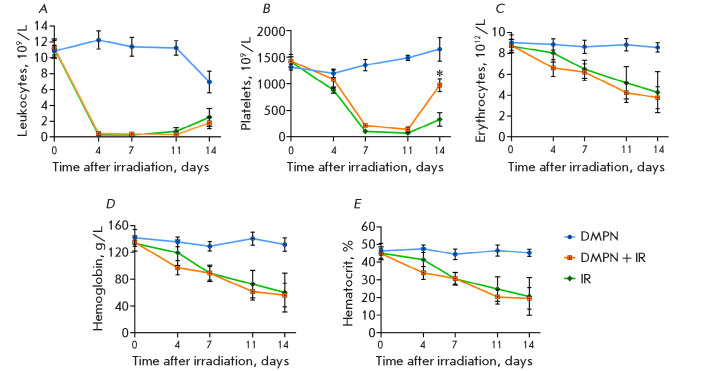
The dynamics of the hematological parameters of the blood of CD-1 mice (n = 8, M ± SEM) that received Dimephosphon after total-body irradiation at a dose of 5 Gy. (A) leukocytes; (B) platelets; (C) erythrocytes; (D) hemoglobin; and (E) hematocrit on days 4, 7, 11, and 14 post-irradiation. The statistical analysis was performed using the Mann–Whitney U test, *p < 0.05 compared to IR

The dynamics of hematological parameters were identical across the groups of irradiated animals. On day 4 post-irradiation, mice in both the DMPN + IR and IR groups had acute leukopenia; leukocyte counts started to recover on day 14 in both groups (Fig. 4A). The decline in erythrocyte count was related to a concurrent reduction in the hematocrit and hemoglobin levels on days 4 through 14 (Fig. 4C-E). Platelet count dropped abruptly by day 7 but started to recover on day 14 post-irradiation. Administration of Dimephosphon statistically significantly accelerated only the platelet count recovery in irradiated animals on day 14 (Fig. 4B).

**Histological examination of the internal organs of mice exposed at a dose of 5 Gy radiation and administered Dimephosphon**

**Table 1:** Organ mass index (%) of CD-1 mice after total-body irradiation at a dose of 5 Gy (M ± SEM)

Organ	DMPN	DMPN+IR	IR
Thymus	0.38 ± 0.01	0.15 ± 0.02^*^	0.11 ± 0.03^*^
Heart	0.52 ± 0.02	0.53 ± 0.01	0.46 ± 0.02
Lungs	0.98 ± 0.07	1.05 ± 0.07	0.96 ± 0.05
Liver	5.85 ± 0.27	4.78 ± 0.60	5.34 ± 0.44
Spleen	0.74 ± 0.11	1.18 ± 0.40	1.29 ± 0.23

Note: Statistical analysis was carried out using the Mann– Whitney U test, ^*^p < 0.05 compared to DMPN.

Exposure to X-ray radiation statistically significantly altered the mass index of the thymus (Table 1). The observed acute involution of the thymus (reduction in its mass index more than twofold) in the DMPN + IR and IR groups was probably associated with an abrupt decline in the counts of T-lymphocytes and thymic epithelial cells [23]. The mass ratio of the spleen was also increased in the groups of mice exposed to radiation. However, this effect was statistically non-significant.

**Fig. 5:**
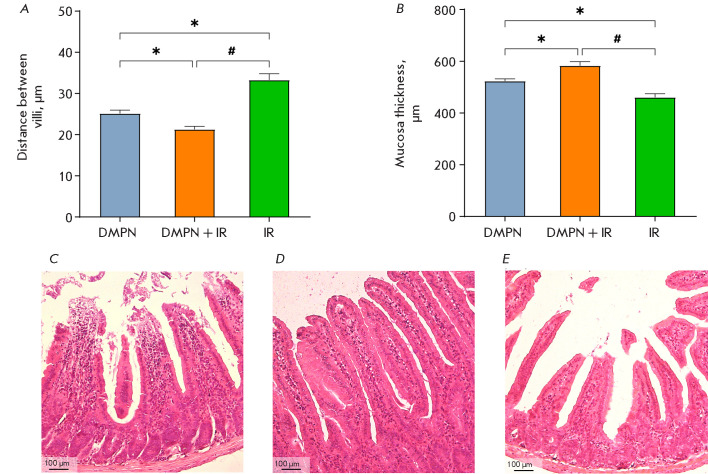
The effect of Dimephosphon on radiation-induced damage to the small intestine of CD-1 mice. (A) The distance between villi and (B) thickness of the small intestine mucosa (M ± SEM). The histological presentation of the small intestine in the groups: (C) DMPN; (D) DMPN + IR; and (E) IR. Hematoxylin and Eosin staining, ×100 magnification. The statistical analysis was performed using the Student’s t test, *p < 0.05 compared to DMPN, #p < 0.05 compared to DMPN + IR

The histological data showed that mice in the DMPN group had a typical structure of the small intestine, with normal length of the intestinal villi and normal crypt depth Fig. 5B. Contrariwise, mice in the IR group suffered radiation-induced injury to the small intestine such as atrophy and shortening of the intestinal villi, along with an increased distance between them (Fig. 5A,E). Furthermore, thickness of the small intestinal mucosa was smaller compared to the DMPN and DMPN + IR groups (Fig. 5B). Administration of Dimephosphon to irradiated mice mitigated the severity of the radiation-induced injury: increased length of the intestinal villi, reduced intervillous space, and greater crypt depth were observed (Fig. 5A,D). Mucosa thickness in the DMPN + IR group was greater than that in the DMPN group, being probably related to enhanced regeneration of the intestinal epithelium (Fig. 5B).

**Fig. 6:**
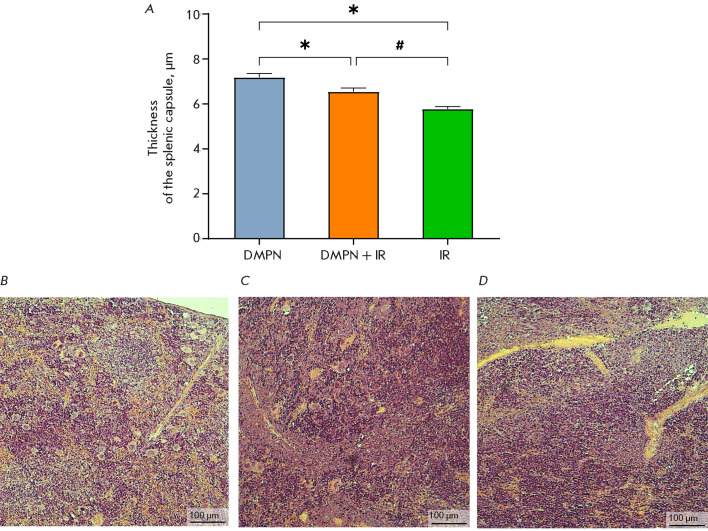
The effect of Dimephosphon on radiation-induced splenic damage in CD-1 mice. (A) Thickness of the splenic capsule (M ± SEM). The histological presentation of the spleen in the groups: (B) DMPN; (C) DMPN + IR; and (D) IR. Hematoxylin and Eosin staining, ×100 magnification. The statistical analysis was performed using the Student’s t test, *p < 0.05 compared to DMPN, #p < 0.05 compared to DMPN + IR

The splenic architecture morphology in the animals in the DMPN group was normal (white and red pulp separated by a marginal zone and covered by a connective tissue capsule) (Fig. 6B). The splenic architecture was altered by day 14 post-irradiation: white pulp had expanded because of cell proliferation, and the distinct boundary between the red and white pulp had disappeared (Fig. 6D). Massive lymphocytic infiltration of the red pulp was observed, with lymphocytes initially residing in splenic sinusoids and ligaments. The sinusoidal spaces were enlarged and had increased blood filling. Furthermore, thickness of the splenic capsule in the IR group was reduced compared to the control group (Fig. 6A), which is consistent with the observed trend toward an elevated mass ratio for this organ (Table 1) and is an indicator of splenomegaly [24]. Administration of Dimephosphon to mice exposed to radiation did not change the architecture of the splenic parenchyma but contributed to a restoration of normal thickness for the splenic capsule (Fig. 6A,C).

Hence, the histological findings give grounds for suggesting that Dimephosphon exerts a local radioprotective effect by mitigating the severity of radiation-induced injury to the small intestine and splenic inflammation.

**Metabolomic screening of the blood samples from mice exposed at a dose of 5 Gy radiation and administered Dimephosphon**

**Fig. 7:**
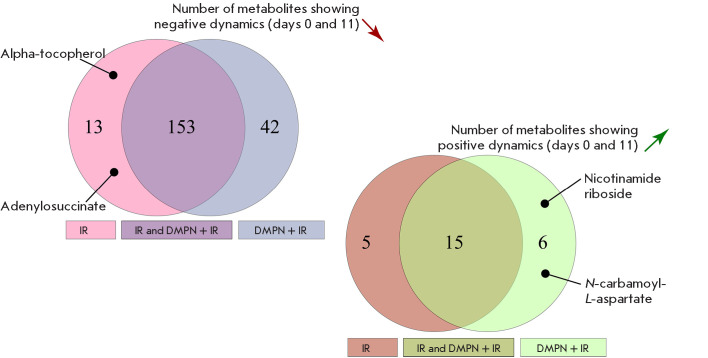
Euler diagrams for metabolites showing the positive and negative dynamics

Metabolomic screening of 489 metabolites was conducted for mice in the DMPN + IR and IR groups at key experimental time points. The statistical analysis algorithm involved metabolite examination between days 0 and 11; among those, metabolites having statistically significant differences were selected. Set intersections were analyzed to identify metabolites unique to each group. A total of 208 metabolites were found to show negative dynamics (153 metabolites exhibited negative dynamics in both groups, 13 metabolites were unique to the IR group, and 42 metabolites were unique to the DMPN + IR group); 26 metabolites showed positive dynamics (15 metabolites exhibited positive dynamics in both groups, 5 metabolites were unique to the IR group, and 6 metabolites were unique to the DMPN + IR group) (Fig. 7).

**Fig. 8:**
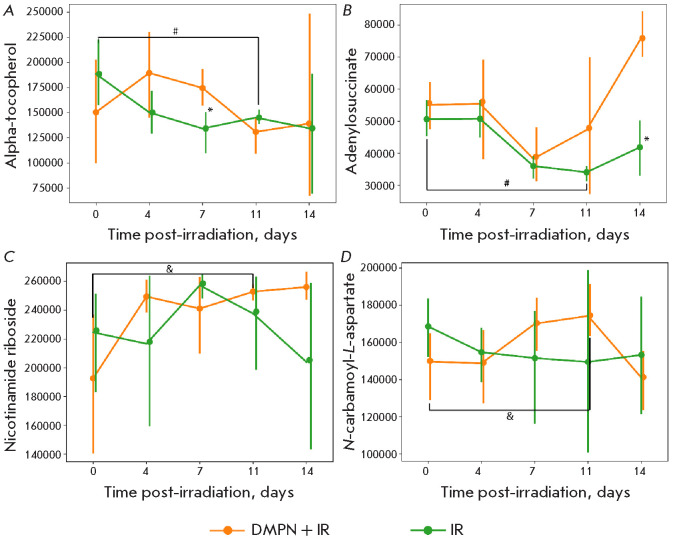
Metabolites characterized by the most statistically significant changes in the blood levels of CD-1 mice in the DMPN+IR and IR groups: (A) alpha-tocopherol, (B) adenylosuccinate; (C) nicotinamide riboside; and (D) N-carbamoyl-L-aspartate. Data are presented as mean ± confidence interval for a nonparametric sample. The statistical analysis was performed using the Mann–Whitney U test, *p < 0.05 between the DMPN + IR and IR groups, #p < 0.05 between days 0 and 11 in the IR group, &p < 0.05 between days 0 and 11 in the DMPN + IR group

After the irradiation, mice in the IR group had abnormal tocopherol metabolism, characterized by a gradual decline in its level throughout the experiment, increasing the organism’s susceptibility to free radical damage. Figure 8A shows that the blood level of tocopherol in the DMPN + IR group was not reduced, as opposed to that in the IR group. Alphatocopherol is a potent fat-soluble antioxidant exhibiting antioxidant and radioprotective effects due to free radical scavenging [25] and the indirect impact on the secretion of specific growth factors and cytokines [26]. Therefore, the observed dynamics of the alphatocopherol level can indirectly attest to the radioprotective mechanism of the drug.

The adenylosuccinate level (Fig. 8B) was decreasing abruptly in both groups until day 7 post-irradiation, followed by a significant rise in the metabolite level in the DMPN + IR group, which was not observed in the IR group. Adenylosuccinate is involved in purine recycling, energy homeostasis, as well as mitigation of inflammation and other types of cellular stress [27]. Importantly, the blood levels of purine metabolites correlate with cellular resistance to radiation; their exogenous administration contributes to the repair of double-strand DNA breaks after exposure to radiation [28]. Hence, the administered drug Dimephosphon compensated for the effects of irradiation by increasing the blood level of adenylosuccinate, which may be indication that the drug can enhance the organism’s resistance to radiation.

The level of nicotinamide riboside remained virtually unchanged after irradiation; a slight rise was observed by day 7. However, administration of the drug abruptly increased the blood level of this metabolite in mice by day 4, which persisted until the end of the experiment (Fig. 8C). Nicotinamide riboside is a precursor of NAD^+^, which acts as a coenzyme for many cellular reactions involved in the physiological homeostasis of various organs and systems. This metabolite was shown to affect the progression of acute radiation syndrome; its oral administration exerts a radioprotective effect by inhibiting cellular senescence in the spleen and normalizing the serum metabolite profile in mice [29]. Furthermore, recent studies have demonstrated that NAD^+^ precursors, and nicotinamide riboside in particular, play a pivotal role in maintaining the integrity of the intestinal barrier [30]. The positive dynamics of the nicotinamide riboside level observed in our study are consistent with the histological data and can explain the radioprotective effect in the small intestine exerted by the drug.

In the IR group, the N-carbamoyl-L-aspartate level remained constant, whereas increased levels were observed on days 7 and 11 after administration of Dimephosphon (Fig. 8D). Cheema et al. [31] reported the level of this metabolite decreasing in mouse intestinal tissues after single-dose total-body gamma irradiation. N-carbamoyl-L-aspartate is an early intermediate in the de novo synthesis of pyrimidines, which is essential for cell proliferation and damaged tissue repair. N-carbamoyl-L-aspartate is formed via the condensation of carbamoyl phosphate and aspartate, catalyzed by aspartate carbamoyltransferase. The elevated N-carbamoyl-L-aspartate level in the DMPN + IR group may attest to an activation of pyrimidine synthesis aimed at epithelial repair and involvement of this compound in the radiation-induced adaptive response. That would be consistent with the fact revealed in our study that the intestinal mucosa was restored after administration of the drug.

In this study, we have revealed alterations in the levels of alpha-tocopherol, adenylosuccinate, nicotinamide riboside, and N-carbamoyl-L-aspartate. The observed differences in their levels between the IR and DMPN + IR groups can be the biochemical markers of the radioprotective efficacy of the drug. These metabolites are involved in the antioxidant processes taking place in the cells; the detected metabolomic changes are indicative of the processes manifesting themselves at the tissue level as reduced severity of injury to the small intestinal mucosa and reduction in the thickness of the splenic capsule.

## CONCLUSIONS

Our findings allowed us to choose the radiation dose and characterize the key indicators of systemic injury to further study potentially promising radioprotective agents. Dimephosphon was also found to mitigate the severity of radiation-induced injury to the small intestinal mucosa and the splenic capsule, as well as contribute to the restoration of platelet counts in CD-1 mice after single-dose irradiation. Meanwhile, the analysis of other key hematological parameters and animal survival rates yielded no evidence of the radioprotective effect of Dimephosphon. The metabolomic data, namely, the significant increase in the blood levels of alpha-tocopherol, nicotinamide riboside, N-carbamoyl-L-aspartate, and adenylosuccinate in mice administered Dimephosphon, are in line with the histological findings for the intestinal mucosa and spleen and demonstrate that Dimephosphon exhibits an antioxidant activity.

## References

[1] Kaprin AD., Starinsky VV., Shakhzadova AO. (2024). State of oncologic care for the Russian population in 2023. P.A. Hertsen MORI – branch of FSBI «NMMRC» of the Ministry of Health of the Russian Federation; 2024..

[2] Martin OA., Martin RF. (2020). Cancer Radiotherapy: Understanding the Price of Tumor Eradication.. Front Cell Dev Biol..

[3] Velsher LZ., Kosmynin AA., Byakhov MYu., Duditskaya TK., Reshetov DN. (2012). Targeted Therapy: A New Approach for the Treatment of Locally Advanced Oropharyngeal Cancer.. Acta Naturae..

[4] Dale DC., Crawford J., Klippel Z. (2018). A Systematic Literature Review of The Efficacy, Effectiveness, and Safety of Filgrastim.. Support Care Cancer..

[5] Lee M., Yee J., Kim JY. (2019). Risk Factors for Neutropenia and Febrile Neutropenia Following Prophylactic Pegfilgrastim.. Asia Pac J Clin Oncol..

[6] Andreassen CN., Grau C., Lindegaard JC. (2003). Chemical Radioprotection: A Critical Review of Amifostine as a Cytoprotector in Radiotherapy.. Semin Radiat Oncol..

[7] Mun GI., Kim S., Choi E., Kim CS., Lee YS. (2018). Pharmacology of Natural Radioprotectors.. Arch Pharm Res..

[8] Raj S., Manchanda R., Bhandari M., Alam MS. (2022). Review on Natural Bioactive Products as Radioprotective Therapeutics: Present and Past Perspective.. Curr Pharm Biotechnol..

[9] Scott BR., Lin Y., Saxton B., Chen W., Potter CA., Belinsky SA. (2021). Modeling Cell Survival Fraction and Other Dose-Response Relationships for Immunodeficient C.B-17 SCID Mice Exposed to 320-kV X Rays.. Dose Response..

[10] Vizel AA., Vizel AO., Shchukina LI. (2013). Dimethyl oxobutylphosphonyl dimethylate (Dimephosphone): use in pulmonology and phthisiology.. Pulmonologiya..

[11] Maksimov ML., Malykhina AI., Shikaleva AA. (2020). Time-tested pharmacotherapy: from mechanisms to clinical efficacy.. RMJ..

[12] Studentsova IA., Danilov VI., Khafizyanova RH. (1995). Results of Clinical Testing of Dimephosphon as a Vasoactive Agent That Normalizes the Functions of the Nervous System.. Kazanskiy Meditsinskiy Zhurnal..

[13] Mironov VF., Buzykin BI., Garaev RS. (2014). Dimephosphone analogs: a pharmacological aspect.. Russ Chem Bull..

[14] Poluektov MG., Podymova IG., Golubev VL. (2015). Possibilities of using the drug dimephosphone in neurology and neurosurgery.. Doctor.Ru..

[15] Gileva TG., Lukin AV., Nyushkin AA., Agachev AR., Studentsova IA., Vizel AO. (1994). Metrology of acute radiation reaction in patients with laryngeal cancer.. Kazanskiy Meditsinskiy Zhurnal..

[16] Li K., Naviaux JC., Monk JM., Wang L., Naviaux RK. (2020). Improved Dried Blood Spot-Based Metabolomics: A Targeted, Broad-Spectrum, Single-Injection Method.. Metabolites..

[17] Basov NV., Rogachev AD., Aleshkova MA. (2024). Global LC-MS/MS Targeted Metabolomics Using a Combination Of HILIC and RP LC Separation Modes on an Organic Monolithic Column Based on 1-vinyl-1,2,4-triazole.. Talanta..

[18] Patrushev YuV., Sotnikova YuS., Sidel’nikov VN. (2020). A Monolithic Column with a Sorbent Based on 1-Vinyl-1,2,4-Triazole for Hydrophilic HPLC.. Prot Met Phys Chem Surf..

[19] Yuan M., Breitkopf SB., Yang X., Asara JM. (2012). A Positive/Negative Ion-Switching, Targeted Mass Spectrometry-Based Metabolomics Platform for Bodily Fluids, Cells, and Fresh and Fixed Tissue.. Nat Protoc..

[20] Li K., Naviaux JC., Bright AT., Wang L., Naviaux RK. (2017). A robust, single-injection method for targeted, broad-spectrum plasma metabolomics.. Metabolomics..

[21] Tairbekov MG., Petrov VM. (2005). Medical-biological effects of ionizing radiation. Moscow: MEPhI. 2005..

[22] Macià I., Garau M., Lucas Calduch A., López EC. (2011). Radiobiology of the Acute Radiation Syndrome.. Rep Pract Oncol Radiother..

[23] Horie K., Namiki K., Kinoshita K. (2023). Acute Irradiation Causes a Long-Term Disturbance in the Heterogeneity and Gene Expression Profile of Medullary Thymic Epithelial Cells.. Front Immunol..

[24] Tripathi AM., Khan S., Chaudhury NK. (2022). Radiomitigation by Melatonin in C57BL/6 Mice: Possible Implications as Adjuvant in Radiotherapy and Chemotherapy.. In Vivo..

[25] Tucker JM., Townsend DM. (2005). Alpha-tocopherol: Roles in Prevention and Therapy of Human Disease.. Biomed Pharmacother..

[26] Singh VK., Beattie LA., Seed TM. (2013). Vitamin E: Tocopherols and Tocotrienols as Potential Radiation Countermeasures.. J Radiat Res..

[27] Rybalka E., Kourakis S., Bonsett CA., Moghadaszadeh B., Beggs AH., Timpani CA. (2023). Adenylosuccinic Acid: An Orphan Drug with Untapped Potential.. Pharmaceuticals (Basel)..

[28] Zhou W., Yao Y., Scott AJ. (2020). Purine Metabolism Regulates DNA Repair and Therapy Resistance in Glioblastoma.. Nat Commun..

[29] Li W., Wang X., Dong Y. (2023). Nicotinamide Riboside Intervention Alleviates Hematopoietic System Injury of Ionizing Radiation-Induced Premature Aging Mice.. Aging Cell..

[30] Niño-Narvión J., Rojo-López MI., Martinez-Santos P. (2023). NAD+ Precursors and Intestinal Inflammation: Therapeutic Insights Involving Gut Microbiota.. Nutrients..

[31] Cheema AK., Suman S., Kaur P., Singh R., Fornace AJ Jr., Datta K. (2014). Long-Term Differential Changes in Mouse Intestinal Metabolomics after γ and Heavy Ion Radiation Exposure.. PLoS One..

